# Knowledge and preparedness for home-based, family-centered management of COVID-19 patients and dead bodies among residents in a COVID-19 high-risk setting

**DOI:** 10.21203/rs.3.rs-942168/v1

**Published:** 2021-09-28

**Authors:** Blaise Kiyimba, Teddy Onyait, Livingstone Kamoga, Arnold Atuhaire, Patrick Ssekatono, Lucy Mujawamariya, Ronald Olum, Felix Bongomin, Sarah Kiguli

**Affiliations:** Makerere University Faculty of Medicine: Makerere University College of Health Sciences; Makerere University Faculty of Medicine: Makerere University College of Health Sciences; Makerere University CHS: Makerere University College of Health Sciences; Makerere University Faculty of Medicine: Makerere University College of Health Sciences; Makerere University CHS: Makerere University College of Health Sciences; Makerere University CHS: Makerere University College of Health Sciences; Makerere University Faculty of Medicine: Makerere University College of Health Sciences; Gulu University; Makerere University Faculty of Medicine: Makerere University College of Health Sciences

**Keywords:** COVID-19, home-based care, dead bodies, Uganda

## Abstract

**Background:**

The overwhelming coronavirus disease- 2019 (COVID-19) cases have called for inevitable home-based care for some cases and direct involvement in COVID-19 dead body burials by many families worldwide. However, data on the knowledge and readiness by families for these practices is still scarce, hence this study among residents of Wakiso district, Uganda.

**Methods:**

We conducted a cross-sectional study between 6th March and 4th April 2021. Household heads aged 15years and above in 5 sub counties of Wakiso district were interviewed using a pre-tested questionnaire. Multivariable logistic regression analysis was used to assess the association between COVID-19 related knowledge on home-based care and burials with demographics characteristics.

**Results:**

We enrolled 205 participants, with a median age of 28 (range: 25–35) years. Majority (n = 157, 76.6%) were female and had achieved at least secondary level of education (n = 117, 57.1%). The mean knowledge score on home-based care for COVID-19 patient was 49.5 %, while that on COVID-19 dead body management was 36.5%. Seven (3.4%) respondents were ready to undertake home-based care and dead body management. For the remainder 198 (96.6%) unready respondents reported inadequate knowledge (n = 166, 84%) and lack of personal protective equipment (PPE) (n = 17, 8.6%) as major barriers for their readiness. There was no statistically significant difference in both the knowledge on home-based COVID-19 patient care and dead body management stratified by demographics characteristics.

**Conclusion:**

The knowledge and preparedness for home-based Covid-19 patient care and dead body management are suboptimal among Wakiso district residents. More public education programmes and PPE provision are recommended.

## Introduction

The world is experiencing one of its ever-worst moments- the coronavirus disease-2019 (COVID-19) pandemic. The outbreak started in Wuhan City China in the late 2019, and was declared a global emergency on January 30th and a pandemic on 12th March 2020 [1, 2]. COVID-19 is caused by the novel severe acute respiratory syndrome coronavirus-2 (SARS-CoV-2) [3] and it is associated with respiratory, intestinal [4], mental, and neurological [5] manifestations. The high contagiousness and explosive spread of the infection has been a matter of concern because it has overwhelmed even the most resilient health systems [6] and has exerted a huge toll on individuals, families, communities, and societies across the world [6].

With the everyday exponential increases in the number of new infections, countries globally especially the developing countries of Sub-Saharan Africa (SSA) and Asia have been overburdened with patients’ numbers beyond the carrying capacity of the existing hospitals [7–11]. In such instances, the World Health Organization (WHO) recommends such overwhelmed countries to employ home-based management of mild and moderate COVID-19 patients to reserve hospital space for only those with severe and critical disease [12] However, existing data shows that for every 100 COVID-19 patients, about 40% of cases will experience mild disease, 40% moderate disease, 15% severe disease, and 5% of cases will have critical disease [6]. With this option implemented, it is evident that about 80% of cases were to be under home- based health care. It is therefore critical to ensure that caregivers have appropriate knowledge on how to care for patients, how to minimize the risk of infection transmission, and on recognising the early signs of a worsening disease condition for urgent transfer to a health facility [12].

As of 26th September 2021, a total of 232,360,218 COVID-19 cases and 4,758,748 deaths had been reported world-wide [13]. In Uganda a total of 123,066cases with 3,143 deaths had been registered [14]. With this overwhelming number of COVID-19 patients and deaths, the country’s national referral hospital declared consideration of home-based management of mild and moderate COVID-19 patients in order to spare hospitals’ space for only the severe and critical covid-19 admissions [10, 15]. In addition, from 10th November 2020, the Uganda ministry of health (MOH) declared overwhelmed with COVID-19 burial costs and allowed families of those who have died of covid-19 to take charge of burying their dead bodies [16]. Studies report that SARS CoV-2 can remain infectious on inanimate surfaces at room temperature for up to 9 days [17] and there is a possibility that the virus also remains on dead bodies [18]. Though the likelihood of covid-19 transmission when handling the dead and their remains is predicted to be low [1, 5, 18–19], the safety and well-being of those involved in managing covid-19 dead bodies is of paramount importance and such people should adhere to the latest safety precautions recommended by their national and the WHO guidelines [18, 20–21] to minimize such transmissions.

Little is known of how much knowledgeable the public- “the now home nurses” is concerning the guidelines of managing COVID-19 patients and dead bodies through burial. Though many COVID-19 awareness campaigns in the country have focused on public transmissions, little efforts have been directed towards home based patient care and dead body management safety precautions. With the already existing high number of professional health care workers dying of COVID-19 globally [22–23] worse COVID-19 death surges would be anticipated from home-based front liners with barely any basic medical safety knowledge.

Currently, there is minimal literature about the public’s knowledge stand regarding safe home-based COVID-19 patient care and dead body handling. This study therefore intended to assess the knowledge and preparedness for home-based COVID-19 patient care and family-centred dead body management among residents of Wakiso district which could unveil any existing gaps for appropriate evidence-based interventions that could help prevent avoidable COVID-19 transmissions, reduce new COVID-19 cases, and deaths.

## Materials And Methods

### Study design and area:

This was a cross-sectional study using quantitative techniques conducted between 6th March and 4th April 2021 in Wakiso district Uganda.

Wakiso district lies in the central region of the country, bordering with Nakaseke and luweero districts to the north, Mukono district to the east, Kalangala district and Lake Victoria to the south, Mpigi district to the southwest and Mityana district to the northwest. Wakiso district head-quarters are located approximately 20 kilometres northwest of Kampala, the capital of Uganda. It is the second most populated district in the country and was selected as our study site because it ranked second after Kampala among districts reporting the highest number of COVID-19 patients and deaths in the country.

The district consists of 17 sub counties, with a total population of 1,997,418 people and 501467 households (2014 national census results).

### Study population:

We conducted this study among male and female residents of Wakiso district Uganda, aged 15 years and above. We included participants below 18 years of age because there was a significant number of household heads in this district who were less than 18 years, hence their opinions too had to be equally considered. The target population were the household heads in Nangabo, Wakiso, Nsangi, Nabweru, and Wakiso town council sub counties of wakiso district Uganda.

### Sample size and sampling:

The sample size was calculated using Cochran’s equation for sample size estimation:

n=Z^2•PQ/e^2

Where n = sample size, Z (z-value at confidence interval 92%) = 1.75, P (predicted existing prevalence of knowledge and preparedness) = 40%, Q (100-P) = 60% and e (precision level) = +/−06%, n = 205 household heads.

We obtained 41 participants from each of the selected sub counties mentioned above using simple random sampling of the households.

### Selection criteria:

Household heads in the selected sub counties of wakiso district who were of sound mind and who consented to participate in the study were recruited. Household heads who were not available at their homes at the time of data collection and those who by the investigators’ judgement were severely ill to respond to the questions were excluded.

### Study Variables:

Independent variables included sex, age, and maximum level of education. Dependent variables included knowledge on covid-19 patient care, covid-19 dead body management, and family preparedness.

### Questionnaire development:

Since we did not find any pre-validated questionaire used in previous studies about the same topic, we developed the questionnaire using the “WHO Home care for patients with suspected or confirmed Covid-19 and management of their contacts guidelines: interim guidance 12 August 2020” [12] and the “WHO Infection prevention and control for the safe management of a dead body in the context of covid-19: Interim guidance. 2020 September” [5]. It comprised of four sections:

1. Three questions on socio-demographic characteristics;
2. Nine questions on knowledge about home-based Covid-19 patient care;
3. Nine questions on knowledge about covid-19 dead body management;
4. Two questions about the preparedness for home-based Covid-19 patient care and Covid-19 dead body management.

The questions under each section were developed by the authors.

### Quality assurance:

The research assistants underwent an extensive training about the research study in order to minimise possible errors during data collection. The questionnaire was pre- tested on 10 household heads in two of the non-selected sub counties of Wakiso district and all the necessary corrections required were noticed and addressed before administering the tool to the final study participants. Each questionnaire used per participant was cross-checked for completeness at the end of the interview. At the end of the data collection period, all the collected data was entered into EpiCollect data forms and was always re-checked by the principal investigator for accurate entries.

### COVID-19 SAFETY PRECAUTIONS

The study was conducted while following the MOH and the Uganda COVID-19 Task Force guidelines. We provided free medical face masks to all data collectors and the selected study subjects. Each data collector was in addition given a personal hand sanitiser. Prior to each interview conducted, both the interviewer and the interviewee were required to first wear the provided face mask, hand sanitise, and stand/sit at a distance of at least 1meter during the interview.

### Data collection process

We used an interviewer- administered questionnaire translated to *Luganda*- the local language in the district, for effective understanding and appropriate answering of the questions. By simple random sampling of households in each of the selected sub counties, a selected household head was approached through a home visit by the data collectors. While observing COVID-19 standard operating procedures (SOPs), a brief introduction about the study was given followed by obtaining informed consent, and answering the questionnaire. The data was collected during a period of 5 weeks, done on only Saturdays and Sundays to ensure maximum capturing of household heads when they were not at their respective work places

### Data analysis

Data was extracted from EpiCollect, cleaned and coded using Microsoft Excel 2016. Statistical analysis was performed using STATA 16 (College Station, Texas, USA). At univariate analysis, all categorical variables were presented as frequency and percentages whereas numerical variables like age was presented as median and interquartile ratio (IQR) since it was non-parametric. The knowledge on home-based was assessed using 9 questions but each participant could score a maximum of 20 points. The individual scores were then converted to percentages. Knowledge on burial of COVID-19 deceased persons was calculated from nine questions but each participant could score a maximum of 12 points. Each correct response was awarded one point and thereafter individual scores calculated by totalling scores from the nine responses and presented as a percentage. A total score above 50% was considered good knowledge while that below 50% low knowledge. Linear regression was performed to assess the association between demographic variables and COVID-19 related knowledge on both home-based management and burial of the deceased. Results were presented as crude co-efficient, adjusted co-efficient, 95% confidence intervals and p-values. A p < 0.05 was considered statistically significant.

## Results

### Demographics

The total number of participants enrolled in this study was 205 people. Their median age was 28 (interquartile range: 25–35) years. The majority (n = 90, 43.9%) of the participants were between the age of 26 and 35 years, were female (n = 157, 76.6%) and had secondary level of education (n = 117, 57.1%). Table 1 summarizes the sociodemographic characteristics of the participants.

#### Knowledge on home-based care of patients with COVID-19

The mean knowledge score on home-based care of COVID-19 patients was 49.5%. Seventy-four (36.1 %) participants knew that a medical mask is the recommended facemask to be worn by COVID-19 patients’ caretakers (Table 2). However, 200 (97.6%) knew that both gloves and a mask must be worn when handling patient’s body fluids such as blood, sweat, saliva, stool, and urine. The average knowledge about early warning signs for worsening COVID-19 disease was 18.28%, with difficulty in breathing being the most known warning sign (n = 61,29.8%). One hundred and fifty-four (74.6%) participants knew that a COVID-19 patient must be limited to only one room, with only one caretaker assigned to them (n = 195, 95.1%), and without hosting any visitor at home until the patient has fully recovered (n = 191, 93.2%). People above 60 years were the most known category of persons at high risk of getting severe form of COVID-19 (n = 155, 75.6%). Ninety-two (44.9%) and thirty-two (15.6%) of participants knew all the three definitions of a COVID-19 contact and the recommended steps of cleaning surfaces exposed to a COVID-19 patients respectively. With regards to handling belonging of patients, majority of participants knew that utensils (n = 138,55%) and clothing (n = 121,59%) can be re-used by the patient. Overall, 177 (57.1%) participants scored above the mean mark for home-based care of COVID-19 patients.

#### Knowledge on COVID-19 Dead Body Management

The mean knowledge score on COVID-19 dead body management was 36.5%. Though majority (n = 170, 82.9%) knew the right thing to be done by the first person to encounter a COVID-19 dead body, only 43 (21.0%) knew the most suitable equipment for protection against the dead body’s fluids splashes. One hundred sixty-six (81.0%) participants didn’t know the recommended number of people for active preparation of the dead body for burial while 137 (66.8%) believed that cultural practices such as bathing the dead body, trimming hair and cutting nails must not be done on COVID-19 dead bodies (Table 3). Overall, 93 (45.4%) participants scored above the mean mark for dead body management.

### Preparedness

Of the 205 respondents, only 7 (3.4%) felt they were ready to participate in safe home-based care of COVID-19 patients and safe burial arrangements as a family. Three of these reported to have undertaken a special training in doing so. Of the 198 (96.6%) participants not ready to participate in home-based care,167(84%) reported inadequate knowledge as the reason. Lack of supplies like PPE (n = 17, 8.6%), infectious nature of SARS-CoV-2 (n = 12, 6.0%) and insufficient space at home (n = 6, 3.0%) were also stated. Similarly, insufficient knowledge (n = 173, 87.4%) was still the most frequent reason for unreadiness to undertake COVID-19 dead body burial arrangements. Other reasons included fear of getting infected with SARS CoV-2 (n = 10, 5.1%), lack of PPE required for burial (n = 16, 8.0%) and lack of space (n = 1, 0.52%).

Both the knowledge on home-based care of COVID-19 patients and dead body management did not significantly differ by age, sex or level of education at both simple linear regression and multivariable logistic regression analyses after adjusting for all the three demographic variables (Table 4). There was also very weak correlation between knowledge on COVID-19 home-based care and dead body management but this was not statistically significant (Spearman’s Rho = 0.03, p = 0.645).

## Discussion

The on-going COVID-19 pandemic is still a global puzzling jeopardy whose fate has remained unknown yet. The overwhelming patient numbers at most health facilities accompanied by the still much fear for stigma from the public among those infected [24–27] have forced many to adopt home-based isolation and management of their mild to moderate symptoms, accepting hospital admissions only after progressing to severe symptoms. Sufficient knowledge and strict implementation of the recommended safe home-based COVID-19 patient care and dead body management guidelines in the general population are therefore key to minimise chances of further spread of the disease among family members and the community.

To our knowledge, little data exists globally concerning the knowledge and readiness for COVID-19 home-based care and dead body management. In this study, we were able to reveal that the knowledge level about both home-based COVID-19 patient care and dead bodies’ management was below 50%, and that over 90% of the residents were not ready to undertake those two initiatives.

This study found out that the mean knowledge about home-based covid-19 patient care among participants was at 49.5%, which is a low score. It could be because by the time the study was done, no awareness communications specifically about home-based Covid-19 patient care had been extended to communities in the country. This finding was congruent with that reported among patients at a public hospital in Uganda by Twinamasiko and others which showed a general knowledge about COVID-19 at only 50% [28]. However, it is in contrast to the study done by Zhong and colleagues among Chinese residents whose covid-19 knowledge score was as high as 90% [29] and the study by Geldsetzer P among United States (US) and United Kingdom (UK) residents, which showed a generally good knowledge [30]. The reason for this big difference in the knowledge between Ugandan residents and those of UK, US, and China could possibly be due to the relatively very big number of covid-19 cases received by the latter nations which could have triggered more self-responsibility and curiosity among their residents to know more about the virus as everyone sensed being a potential candidate for the disease compared to Uganda with comparably lower covid-19 cases. It could also be due to the differences in the extent and forms of awareness strategies employed by the respective countries.

In this study, the very low average knowledge (18.28%) expressed about the early warning signs of severe COVID-19 puts many home-cared covid-19 patients at a high risk of progressing from mild-moderate to severe and critical conditions without the care takers’ notice, hence delayed transfer to hospital for advanced management, increasing chances of dying from homes. This low knowledge could possibly be due to the COVID-19 public sensitization message in the country whose content is generally richer in mild to moderate COVID-19 symptoms such as cough, sneezing, body weakness, among others and less emphasis put on severe disease symptoms such as blue lips, confusion. In addition, the message is usually conveyed in English which may not be easily understood as effective as local languages and is via televisions and radios which may not be owned by all the people in communities. This score is however higher than the 5% reported on knowledge about signs of severe pneumonia among care takers in Ghana in 2011[31] but lower than that reported (84%) in the same country later after employing some two community-based strategies [32] Similarly, a small portion (45%) of participants who knew all the clear definitions of a covid-19 contact is worrying because failure to quickly identify such people can spread the virus among family members and the community. We recommend more education emphasis along those lines by the MOH and the covid-19 task force. On the other hand, the good knowledge expressed by majority of participants (> 74%) regarding patient isolation principles such as limitation to only one room, assigning only one care taker, no hosting visitors and a must use of personal protective equipment when handling a patient’s body fluids shows some significant positive impact of the Covid-19 awareness campaign done so far. It is in agreement with a study by Olum and colleagues among Ugandan health care workers [33]. We recommend continuous delivery of public messages containing such content to act as reminders for their actual implementation.

The mean knowledge score concerning safe COVID-19 dead body management among study participants was 36.5%. Though the possibility of transmission of the virus from a covid-19 copse to living humans is still unclear [1, 5, 17–19], this was a low knowledge level characterised by very few participants capable of identifying the most effective PPE to use under different moments of corpse handling such as protection against the dead body’s fluids, and the recommended number of people to prepare the body. In the event that covid-19 dead bodies are infectious, it puts a big infectivity risk in the current settings where families have to directly involve in COVID-19 dead body management and burials of their relatives without any assistance by the MOH personnel. This is still mostly due to insufficient communication messages specific to safe COVID-19 dead body handling by families.

This study also found out that 96.6% of the residents were not ready to undertake both home-based COVID-19 patient care as well as family-centred COVID-19 dead body burial arrangements. 84% of these reported insufficient knowledge regarding the proper conduction of such activities as the major block. This shows that despite the little knowledge they have so far, they are not confident enough to perform such activities as families alone. Of the 3.4% (n = 7) respondents who were ready to perform such activities, 3 were health care workers who reported to have undertaken a special training in doing so. This could imply that if such similar training is provided to the general public at large, the percentage of those ready to take on this proposed new involvement would increase, with reduced fear and chances of viral transmissions among people in homes. This is congruent with an observation by Sharma et al in India where family members of a covid-19 deceased refused to even have a look at the dead body due to fear [34].

In this study, there was no association between the residents’ knowledge on home-based patient care, dead body management or preparedness with age, sex, or education level. This could possibly be due to the fact that COVID-19 is a new issue which came with almost uniform sensitization to the public regardless of one’s sex, age or education level hence uniform exposure to covid-19 information and roughly equal knowledge acquisition by all groups of people.

This study has some limitations. Firstly, no standardised tool had been previously validated for assessment of knowledge on COVID-19 home-based care, dead body management or their preparedness. However, the questions have been formulated based on the WHO guidelines for home-based COVID-19 patient care and dead body management [5, 12]. Secondly, only residents in one district were surveyed and the results of this study may not reflect the knowledge and preparedness for the entire country. However, being the first study about this in the country, these results can be used to formulate countrywide public education schedules related to covid-19 home-based patient care and dead body management. A similar study may be extended to other districts and countries.

## Conclusion

In this study, knowledge on both home-based care of COVID-19 patient and handling of dead bodies of those who succumbed to the disease was suboptimal. Insufficient knowledge, inadequate PPE, small space at home, and fear of the infectious nature of the virus are the major barriers for the preparedness. Nation-wide public education programmes coupled with sensitization and awareness about home-based COVID-19 patient care and dead body management plus provision of basic PPE to homes by the concerned stakeholders are recommended to address these challenges even in case of future pandemics.

## Figures and Tables

**Table 1:** Characteristics of Participants

Demographics	Frequency	%
**1.Age in years (median, IQR)**	28	25–35
15–25	64	31.2
26–35	90	43.9
36–45	29	14.1
46–60	21	10.2
>60	1	0.5
**2. Sex**		
Female	157	76.6
Male	48	23.4
**3. Highest level of education**		
Primary	68	33.2
Secondary O-level	99	48.3
Secondary A-level	18	8.8
Technical	12	5.9
University	8	3.9

**Table 2:** Participants’ knowledge on home-based covid-19 patient care

Questions	Frequency	%

**1. While caring for a Covid-19 patient, the care taker is recommended to put on**:		

A fabric or cloth face mask	70	34.2

I don’t know	9	4.4

Medical mask	74	36.1

N95 mask	52	25.4

**2. Which of the following symptom(s) in a covid-19 patient Will require urgent calling of a health worker?**		

Light headedness	36	17.6

Blue lips	27	13.2

Chest pain	18	8.8

Difficulty in breathing	61	29.8

Confusion	45	22.0

Cough	32	15.6

I don’t know	3	1.5

**3.During care for a Covid-19 patient at home:, tick where applicable**:		
**a). Patient can move to any room in the house as long as they don’t get out of the house.**		

FALSE	153	74.6

TRUE	51	24.9

I don’t know	1	0.5

**b). Only one care taker should be assigned and all other family members should always avoid entering the patient’s room.**		

FALSE	9	4.4

TRUE	195	95.1

I don’t know	1	0.5

**c). Visitors should not be allowed in home until the patient has fully recovered**		

FALSE	14	6.8

TRUE	191	93.2

**d).Both the gloves and a mask are a must when handling patient’s blood, sweat, saliva, stool, and urine.**		

FALSE	4	2.0

TRUE	200	97.6

I don’t know	1	0.5

**4. Who of the following are at high risk of developing severe covid-19, hence will require closer monitoring?**		

HIV Patients	107	52.2

Patients above 60 years	155	75.6

Former malaria patients	21	10.2

Cancer, diabetes and hypertension patients	111	54.2

I am not sure	4	2.0

**5. How many days should a person positive for Covid-19 but asymptomatic spend in isolation?**		

7	14	6.8

10	2	1.0

14	169	82.4

I don’t know	18	8.8

No need for isolation	2	1.0

**6. How many days should a symptomatic patient and Covid-19 positive spend in isolation?**		

10	2	1.0

14	87	42.4

20	42	20.5

I don’t know	74	36.1

**7. Which of the following people will require to go in isolation (considered a Covid-19 contact)?**		

A person in contact with a COVID-19 ppatient 2 days before or 24 days after the patient tested positive for Covid-19.	27	13.2

One who gets face to face contact with a Covid-19 patient within a less than 1m distance for at least 15 minutes?	18	8.8

Anyone who gets in direct contact with a confirmed Covid-19 patient without recommended protective gears.	52	25.4

All the above.	92	44.9

None of the above	16	7.8

**8. The recommended steps for cleaning surfaces in contact with a Covid-19 patient is?**		

Clean with Jik first, then with soap/detergent, rinse with plain water.	148	72.2

Clean with plain water first, then with soap/detergent, rinse with clean plain water.	19	9.3

Clean with soap/detergent first, then with Jik, rinse with clean plain water	32	15.6

I am not sure	6	2.9

**9. Regarding handling patient’s materials** :		
**a). Patients plates and cups must be washed with soap, Jik, and water**:		

FALSE	38	18.5

TRUE	164	80.0

I don’t know	3	1.5

**b). patient’s utensils must be disposed after eating and never re-used by the patient or someone else.**		

FALSE	138	67.3

TRUE	64	31.2

I don’t know	3	1.5

**c). Patient’s clothes must be buried or burnt and never re-used by the patient or anyone else.**		

FALSE	121	59.0

TRUE	82	40.0

I don’t know	2	1.0

**Table 3:** Participants’ knowledge on management of COVID-19 dead bodies and preparedness

Questions	Frequency	%

**1. Before handling anything on a covid-19 dead body, the very first person to see that body must first**:		

Cover the body with plastic/cloth sheet.	170	82.9

Make the body lie upside down	6	2.9

Remove the mask from the dead	5	2.4

I don’t know	24	11.7

**2. Where splash of body fluids from a Covid-19 dead body is anticipated, one most protective equipment to use is?**		

Face shield	43	21.0

Gloves	97	47.3

N95 mask	37	18.1

Soap and Jik	28	13.7

**3. When aerosols from a Covid-19 dead body are anticipated, the most protective equipment to use is?**		

Face shield	63	30.7

Goggles	3	1.5

I don’t know	9	4.4

Medical mask	81	39.5

N95 mask	49	23.9

**4. Where it is impossible to put a cloth sheet on the Covid-19 dead body, the best alternative is to?**		

I don’t know	52	25.4

Put a medical mask on the dead body	73	35.6

Put a non-medical mask on the dead body	8	3.9

Put on gloves and wash the body	72	35.1

**5. Cultural practices such as trimming nails, shaving hair, and washing the body must never be done on a covid-19 dead body.**		

FALSE	64	31.2

TRUE	137	66.8

I don’t know	4	2.0

**6. The recommended number of people to actively prepare the Covid-19 dead body for burial (Assistants exclusive) is?**		

1	11	5.4

2	39	19.0

4	119	58.1

Any number	11	5.4

I don’t know	25	12.2

**7. Regarding the Covid-19 deceased’s clothings;**		
**a). Clothes must first be soaked in Jik for at least 30 minutes if they are to be washed by hands**		

FALSE	103	50.2

TRUE	86	42.0

I don’t know	16	7.8

**b). They can be reused by other people if well treated.**		

FALSE	115	56.1

TRUE	82	40.0

I don’t know	8	3.9

**c). Clothes must be machine washed if to be used by other people.**		

FALSE	94	45.9

TRUE	95	46.3

I don’t know	16	7.8

**d). Clothes must be burnt or buried.**		

FALSE	79	38.5

TRUE	118	57.6

I don’t know	8	3.9

**8. The shortest distance one must stand at while viewing a covid-19 dead body that has been prepared for public viewing prior to burial is?**	(Median, IQR) 4 meters	Two to Five

**9. Anyone can be allowed to view the prepared covid-19 dead body as long as they don’t touch it**		

FALSE	168	82.0

TRUE	24	11.7

I don’t know	13	6.3

**PREPAREDNESS**:		

**1. Currently, do you feel you are ready to take on all the necessary safe precautions forhome-based Covid-19 patient care instead of hospital-based in case one of your family members tests Covid-19 positive?**		

No., Reason............	198	96.6

Yes. Reason...........	7	3.4

**2. Do you feel you are ready to take on all the safe burial arrangements as a family in case any of your family members dies of Covid-19?**		

No., Reason...............	198	96.6

Yes. Reason..............	7	3.4

**Table 4:** Linear regression models showing factors associated with knowledge of participants on home-based Covid-19 patient care

Variable	Crude Co-efficient	P	Adjusted Co-efficient	P
**Knowledge on home-based covid-19 patient care**				
**Age**	0.05 (−0.12 to 0.21)	0.523	0.06 (−0.10 to 0.23)	0.456
**Sex**				
Female	1.00		1.00	
Male	0.50 (−3.20 to 4.20)	0.790	−0.34 (−4.37 to 3.68)	0.867
**Education**				
Primary	1.00		1.00	
O-level	0.16 (−3.39 to 3.70)	0.931	0.42 (−0.321 to 4.04)	0.820
A-level	−0.73 (−6.70 to 5.24)	0.810	−0.76 (−6.76 to 5.25)	0.804
Technical	−0.17 (−7.22 to 6.88)	0.962	−0.06 (−7.15 to 7.03)	0.986
University	4.41 (−4.01 to 12.83)	0.303	4.94 (−4.17 to 14.06)	0.286
**Knowledge on COVID-19 dead body management**				
**Age**	0.00 (0.00 to 0.00)	0.427	0.00 (0.00 to 0.00)	0.584
**Sex**				
Female	1.00		1.00	
Male	−0.01 (−0.05 to 0.04)	0.686	−0.01 (−0.06 to 0.04)	0.755
**Education**				
Primary	1.00		1.00	
O-level	−0.02 (−0.06 to 0.02)	0.313	−0.02 (−0.06 to 0.02)	0.380
A-level	0.01 (−0.06 to 0.08)	0.728	0.01 (−0.06 to 0.09)	0.725
Technical	−0.02 (−0.10 to 0.07)	0.725	−0.01 (−0.10 to 0.07)	0.749
University	−0.03 (−0.13 to 0.07)	0.527	−0.02 (−0.13 to 0.09)	0.666

**Abbreviations:** IQR, interquartile range; %, percentage; >, greater than; Covid-19, Corona virus disease 2019; P, probability
